# The *West Riding Lunatic Asylum Medical Reports*: the precursor of *Brain*?

**DOI:** 10.1093/brain/awad219

**Published:** 2023-07-03

**Authors:** Andrew J Larner

**Affiliations:** Department of Brain Repair and Rehabilitation, Institute of Neurology, University College London, London, WC1E 6BT, UK

**Keywords:** Brain, cerebral localization, Ferrier, Hughlings Jackson, West Riding Lunatic Asylum Medical Reports

## Abstract

First published in 1878, *Brain: A Journal of Neurology* is generally considered to be the world’s first neuroscientific journal. However, this claim might be challenged since the *West Riding Lunatic Asylum Medical Reports*, another journal with significant neuroscientific content, was published between 1871 and 1876. Some have suggested this journal was the precursor of *Brain*, since it shared similar subject matter as well as editorial and authorial contributors, including James Crichton-Browne, David Ferrier and John Hughlings Jackson.

To address this question, this article examines the origins, aims, structure and contents of, and some of the contributors and contributions to, the *West Riding Lunatic Asylum Medical Reports* and compares these elements to the first six volumes of *Brain* (1878–9 to 1883–4). Although the two journals did overlap in terms of some shared neuroscientific interests, *Brain* evidently had a broader scope and a more international authorship.

Nevertheless, this analysis suggests that, through the agency of Crichton-Browne, Ferrier and Hughlings Jackson, it is appropriate to regard the *West Riding Lunatic Asylum Medical Reports* as not only the antecedent but also the precursor of *Brain*.

## Teaser

Andrew J. Larner examines the six volumes of the *West Riding Lunatic Asylum Medical Reports*, published 1871–76, and compares these to the first six volumes of *Brain*, first published 1878, to ascertain whether the former was, as has sometimes been claimed, the precursor of the latter journal.

## Introduction

Every neurologist knows (or certainly should know!) that *Brain: A Journal of Neurology* was first published in 1878. It has been asserted that ‘*Brain* was the world’s first neuroscientific journal’^1^ and the ‘first truly neurological journal published in English’.^2^ However, these accolades might be susceptible to challenge, since another journal, sometimes characterized as the precursor of *Brain*, had appeared earlier in the 1870s: the *West Riding Lunatic Asylum Medical Reports* (*WRLAMR*).

Some accounts of the role that the West Riding Asylum (WRA) located at Wakefield in West Yorkshire played in the origins of British neurology have appeared.^2-10^ However, little dedicated analysis of its ‘house journal’ has been published, indeed, to my knowledge, there is only a single paper specifically devoted to *WRLAMR*, written by Henry Viets and appearing in 1938 in the *Bulletin of the History of Medicine*.^11^ One quite detailed study, which included *Brain*, its precursor and successors has appeared recently,^12^ as well as one examining developing concepts of epilepsy in the 1870s, as reflected in the pages of *WRLAMR*.^13^

The purpose of this article is to consider the origins and aims of *WRLAMR* and give an overview of its structure and contents as well as some of its contributors and their contributions. In light of this information, a comparison is then made with the early issues of *Brain*, in an attempt to address the question of whether or not the former can rightly be considered the precursor of the latter, of whether it was indeed ‘proto-*Brain*’.

### Origins and aims of *WRLAMR*

*WRLAMR* first appeared in 1871, edited by ‘J Crichton Browne’ (*sic*, without hyphen) and published in London by J. & A. Churchill, retailing at 7*s.* 6*d*.

James Crichton-Browne (1840–1938) had been appointed to the superintendency of WRA at Wakefield in 1866 (not 1871, as stated by Viets^11^) at the age of 25. Founded in 1818 as the sixth county asylum in England to be opened following the discretionary County Asylums Act of 1808, WRA was the first public asylum in the West Riding of Yorkshire. County asylums were funded from the public purse (i.e. by the local ratepayers) as part of the Poor Law system, and so accepted only those patients who were unable to pay (‘paupers’), unlike private asylums. The institution at Wakefield was therefore sometimes known as West Riding Pauper Lunatic Asylum. Most of the clinicians working at WRA who subsequently published in *WRLAMR*, including Crichton-Browne, noted their affiliation to be with the ‘West Riding Asylum’, evidently choosing not to use the words ‘Pauper’ or ‘Lunatic’ (the latter had perhaps already acquired pejorative connotations).

From the outset at WRA, under the inaugural superintendency of William Ellis (1780–1839), the system of moral management of the insane, free of mechanical restraints, was implemented. This approach had been pioneered in England at the York Retreat by the Tuke family and in Paris by Philippe Pinel. The policy was largely maintained under successive WRA superintendents (C.C. Corsellis, J.S. Alderson, J.D. Cleaton).^14,15^ The increasing numbers of patients labelled with insanity in the course of the 19th century necessitated several extensions to WRA and eventually, following the compulsory County Asylums Act of 1845, to the establishment of three more institutions, all operating under the name of West Riding Lunatic Asylum, located at Wadsley, Sheffield (Middlewood Hospital; opened 1872), Menston (High Royds Hospital; 1888), and Huddersfield (Storthes Hall Hospital; 1904).

The aims of the *WRLAMR* were stated explicitly in the preface to the first volume by Crichton-Browne (1871; 1:iii–v) in what we might now call a ‘mission statement’. He observed that ‘In the harmless, but vindictive, attacks which have been recently directed against the public lunatic asylums of this country, it has been a frequent charge that no scientific work is accomplished in them’. Accordingly, he noted that ‘a belief is entertained that there has perhaps been some remissness on the part of those engaged in the superintendence of our hospitals for the insane in publishing the results of their observations,’ perhaps in part due to the ‘absence of any immediate stimulus to the arrangement and elaboration of the materials collected, and to the want of any ready channel of exposition’. Hence ‘It is with a view to supplying this deficiency … that the present volume has been projected’.

What prompted Crichton-Browne to pursue what we might now consider a ‘research agenda’ at WRA, rather than confining his energies to administration of the asylum, a demanding enough requirement, as superintendents did elsewhere? The question is apt since after 1845 the national Lunacy Commissioners had discouraged public asylum superintendents from undertaking research projects that might distract them from their administrative duties.^16^ Nevertheless, Crichton-Browne was evidently aware of the possibilities for research presented by the large number of patients under his care at WRA. Writing to Charles Darwin (1809–82) on 1 June 1869, he noted ‘the mass of interesting material which is as it were going to waste … in this huge hospital for want of accurate observation & which might be of immense value’.^17,18^ (Darwin had originally approached Crichton-Browne for information on facial expressions in the insane as part of the research for his book *The expression of the emotions in man and animals*, published in 1872.)

Indeed, some use had already been made of this ‘interesting material’. Clifford Allbutt (1836–1925), then developing his career as a physician in Leeds, a few miles north of Wakefield, had undertaken ophthalmoscopic examinations of patients at WRA in the second half of 1867, following an invitation from Crichton-Browne. This material was included in various papers and in a monograph on ophthalmoscopy published in 1871, which Allbutt dedicated to John Hughlings Jackson (1835–1911) because of the latter’s renowned advocacy for and skill in fundoscopy. Furthermore, Allbutt’s original description of syphilitic periarteritis of the cerebral arteries published in 1868 was, according to his biographer, based on a specimen sent to him from WRA by Crichton-Browne.^19^

The National Hospital for the Paralysed and Epileptic had opened in Queen Square, London, in 1860, but in its early years was essentially a clinical and not a research-oriented institution, despite having Brown-Séquard (1817–94), a noted experimentalist, on the staff, as well as (from 1862) Hughlings Jackson.^20^ It seems unlikely to have provided a model for Crichton-Browne. However, another clinical institution may have influenced his intention to develop WRA as a hub for research. During his training in asylum medicine, Crichton-Browne had visited Paris, thus following in the footsteps of his father, William Browne (1805–85), also a distinguished alienist, who had visited Esquirol, Pinel’s successor, in the summer and autumn of 1832. It would therefore seem inconceivable that Crichton-Browne would not have visited the Salpêtrière, one of the scenes of Pinel’s (mythical) release of the insane from their chains. Although there are few existing details of Crichton-Browne’s visit to Paris, this occurred around 1862–3, timing coincident with the period when Charcot (1825–93) and Vulpian (1826–87) had both been appointed to chief of service positions at the Salpêtrière and were beginning their work with the large patient numbers accommodated there, for the purposes of clinical assessment and subsequent pathological examination, the clinico-anatomical method. Whether these developments influenced Crichton-Browne is uncertain (the psychiatric and neurological services at the Salpêtrière were quite separate^21^), but it was the view of the historian Janet Oppenheim that Crichton-Browne’s ‘choice of Paris influenced the rest of his career, for clinical observation in hospital or asylum wards and the performance of autopsies, on the French model, became Crichton-Browne’s basic approach to psychological medicine’.^16^ Furthermore, inauguration of new medical journals was a method used extensively by Charcot to communicate the findings of research undertaken by himself and his colleagues, beginning with the *Archives de Physiologie Normale et Pathologique* in 1868, although his first journal devoted to neurology, the *Iconographie photographique de la Salpêtrière*, did not appear until 1876.^21^

Another possible, earlier, influence on Crichton-Browne is that of Thomas Laycock (1812–76). As Chair of the Practice of Physic at Edinburgh from 1855 he initiated instruction in medical psychology and mental diseases and was one of Crichton-Browne’s teachers. He was also a friend of William Browne and, like him, had also visited Paris hospitals in the 1830s. Both through his teaching and his writings, such as *Mind and Brain* (1860), Laycock may have stimulated Crichton-Browne’s interests, although he founded no journal. Of note, two of the key subsequent contributors to *WRLAMR* had also been Laycock’s pupils: Hughlings Jackson, at York Medical School in the early 1850s, and David Ferrier (1843–1928) at Edinburgh in the 1860s.^22^

How was the ‘valuable information, hitherto buried in case-books and diaries’ of WRA to be arranged and elaborated for publication when so much time was ‘absorbed in general or fiscal management’ required to run a public lunatic asylum? Crichton-Browne had initiated various actions in the first years of his superintendency at WRA, which helped to address these issues, related to both personnel and facilities.

In addition to the Superintendent, pauper asylums generally employed one or more Assistant Medical Officers (AMOs), junior clinicians building a career in asylum medicine and handling the day-to-day management of the patients. At Wakefield, one of these roles was temporarily filled in 1857 by Henry Maudsley (1835–1918), later to ascend to the pinnacle of the profession and to become a fierce critic of public asylums. In addition to the AMOs, Crichton-Browne initiated a system of employing Clinical Assistants, unpaid as was the case elsewhere (e.g. the London teaching hospitals), usually newly qualified doctors aspiring to a career in asylum medicine who, for the provision of their board and lodging, were ‘to avail themselves of the vast opportunities … to give breadth and precision to our knowledge of mental diseases’. AMOs and Clinical Assistants were to contribute the majority of papers to *WRLAMR*.

As for facilities, a dedicated pathological laboratory had been built and equipped at WRA by 1872–3. The appointment of a full-time pathologist, T.W. McDowall, in 1872, was a stimulus to research in this field. Although McDowall published only one paper in *WRLAMR*, other WRA pathologists were to make extensive contributions: Herbert Coddington Major and William Bevan Lewis, both eventual successors to the superintendency at WRA, authored six and three papers in *WRLAMR*, respectively making Major the most prolific contributor along with Crichton-Browne. Other contributors also listed their affiliations as ‘Pathologist and Assistant Medical Officer, West Riding Asylum,’ such as William Benham and Robert Lawson.

Crichton-Browne was also interested in the possibilities of photography, setting up a photographic studio at WRA around 1870, wherein George Bracey, the asylum’s dispenser, did most of the work, later followed by Henry Clarke. Some photographs of patients were used in *WRLAMR*, for example in Crichton-Browne’s papers on ‘acute dementia’ and chronic mania. A selection of these photographs from WRA, around 40 in total, was sent to Darwin whilst in pursuit of his research for *The expression of the emotions in man and animals* but not used in the final publication. That this was generally known is suggested by John Milner Fothergill’s (1841–88) comment in *WRLAMR*, writing on ‘Cerebral anaemia’, that ‘If a volume of portraits … were submitted to Mr Darwin, that gentleman would experience no difficulty in recognising in the features of some of them the characteristics of the depressing emotions’ (1874; 4:132–3). This work by Darwin was also noted by other *WRLAMR* authors (1876; 6:137 and 146n1).

Much remains unknown about the workings of *WRLAMR*. Even if case material suitable for publication was plentiful (and of course the concept of ‘patient consent’ did not exist at this time), nevertheless funding for the journal would have been an issue. Perhaps some of the savings Crichton-Browne purportedly made elsewhere, as documented in his annual reports to the asylum’s Committee of Visitors, were put towards the journal, as for the pathological laboratory, but little definitive information appears to be available, apart from ‘a note by the asylum’s committee in 1875 that a grant of £40 be made towards the cost of their printing’.^2^

### Structure and contents of *WRLAMR*

*WRLAMR* was published as six annual octavo volumes between 1871 and 1876. Each of the six volumes ran to about 300 pages (1838 pages in total) and contained from 12 to 15 articles of variable length and type. A total of 80 papers was published in all (Supplementary Table 1); some authors^9,16,23^ have stated 79, although the reason for this discrepancy, other than simple arithmetic error, is not clear. Viets^11^ divided these articles into several categories, namely:

- Papers reporting original research done at West Riding (*n* = 62), these subdivided into (a) Clinical (*n* = 24); (b) Therapy (*n* = 12); (c) Pathological (*n* = 14); (d) Experimental, human (*n* = 8); and (e) Experimental, animal (*n* = 4).
- Papers read at West Riding (*n* = 2).
- Papers contributed to the *Medical Reports* (*n* = 16).

There seems to have been no shortage of material. In the preface to the first issue Crichton-Browne noted that the limits originally assigned necessitated the ‘exclusion of several interesting articles’ (1871; 1:v); in the 1873 preface that ‘Five very valuable essays, containing original observations made in the wards of the asylum, have … been regretfully … excluded’ (1873; 3:iv); and in the 1874 preface that five papers were excluded (1874; 4:v). No criteria for selection or rejection are stated.

Of the clinical papers, some were devoted to specific disease categories, most frequently the catch-all ‘insanity’ but sometimes more particular entities such as general paralysis of the insane, mania, dementia or epilepsy.^13^ The interrelation between general paralysis, locomotor ataxy and syphilis was not defined at this time but both Nicol (1871; 1:178–208) and Bevan Lewis (1875; 5:85–104) noted the similarity of spinal cord lesions in general paralysis and locomotor ataxy, although Aldridge reported that in 43 patients with general paralysis undergoing ophthalmological assessment ‘in two cases only was there any sign of syphilis’ (1872; 2:227). Patient-related investigations included analysis of demographic data that could be obtained from the WRA case books (e.g. hourly distribution of mortality), as well as clinical assessment of specific organ systems (e.g. heart) and faculties (colour perception). In an era before lumbar puncture, neurophysiology and neuroimaging, clinical investigations were limited, for example, pupillometry (1872; 2:223–53), dynamometry (1872; 2:17–18) and ‘urinology’ (1874; 4:63–93).

The application of new or emerging investigative technologies comprised a significant component of the contents of *WRLAMR*. Perhaps the most notable of these was the use of the ophthalmoscope, invented by Helmholtz in 1851. Following Allbutt’s contributions to this literature, based in part on observations made on patients at WRA, the application of the ophthalmoscope was most thoroughly explored in three papers by Dr Charles Aldridge, who made observations ‘in mental and cerebral diseases’ (1871; 1:71–128), ‘in general paralysis, after the administration of certain toxic agents’ (1872; 2:223–53), and ‘in acute dementia’ (1874; 4:291–304). Aldridge’s ophthalmoscopic observations were also mentioned in other authors’ papers, for example Milner Fothergill’s essay on ‘cerebral anaemia’ (1874; 4:94–151), wherein he opined that the ‘ophthalmoscope lights up the pathology of cerebral conditions as regards the vascular system very distinctly’ (1874; 4:115). Hughlings Jackson also contributed an ophthalmological report ‘On a case of recovery from double optic neuritis’ (1874; 4:24–9), illustrated with a chromolithograph, and urged the ‘necessity for the routine use of the ophthalmoscope’. A demonstrating ophthalmoscope, rather than a hand-held device, was used by John Hunter Arbuckle in his experimental observations of retinal arteries and veins in rabbits over many hours as they were administered various pharmacological agents then in clinical use, such as nicotine, atropine, hydrate of chloral, amyl of nitrite, and morphia (1875; 5:130–48).

Otolaryngological studies were also pursued, by Lennox Browne (1841–1902; no relation to Crichton-Browne), a noted early practitioner in this discipline who founded the Central London Throat and Ear Hospital in 1874. He published on ‘Othaematoma, or the insane ear’ (1875; 5:149–59) and ‘Laryngoscopic observations in general paralysis’ (1875; 5:271–83), both studies based on cases seen at WRA, probably on the same visit since the reported total number of inpatients (1424) and the gender distribution (female:male = 717:707) is identical in both reports. John C. Galton contributed ‘Notes on the condition of the tympanic membrane in the insane’ (1873; 3:258–72).

Another novel technology featured in several *WRLAMR* publications was the sphygmograph. This device transcribed the patient’s arterial pulse pressure on to paper. Invented and named by Karl Vierordt (1818–84) and later modified by Étienne-Jules Marey (1830–1904) to provide greater sensitivity and detail, the sphygmograph was later adapted by Charcot for use at the Salpêtrière to record tremors.^21^ At WRA, George Thompson pioneered its use (1871; 1:58–70 and 1872; 2:302–6) but tracings were also mentioned and/or illustrated in papers by Milner Fothergill (1873; 3:126; also 1874; 4:117–8, tracings performed by Lauder Brunton), and by Benham in his experimental work examining the therapeutic effect of cold on the head (1874; 4:152–78, at 164–5, experiments VIII and IX), as well as the actions of nicotine (1874; 4:305–17). Despite Thompson’s evident enthusiasm, the value of sphygmographic recordings was not universally acknowledged at this time.^24^

Therapeutic work exploited a variety of medications available from the asylum pharmacy, as well as other interventions. Finn states that 18 out of the 80 *WRLAMR* publications were directly concerned with tests and trials of the dispensary’s supplies, on both patients and animals,^2^ but gives no further breakdown. The pharmacological agents studied included nitrous oxide, ether, morphia, opium, ergot of rye, conia, nitrite of amyl, nicotine, hyoscyamine, and chloral hydrate. Passing references give some indication of local prescribing practices, for example Crichton-Browne apparently recommended Calabar bean in general paralysis (1871; 1:67 and 1874; 4:91) but used physostigma (derived from the Calabar bean) to control wild outbreaks (1874; 4:111) and found belladonna useful only in the early stages of emotional melancholia (1874; 4:148). Of physical treatments, cold in the form of an ice cap was sometimes used although Benham’s experiments found absolutely no change in brain temperature and Crichton-Browne thought it had no beneficial effect in acute maniacal attacks (1874; 4:176). Allbutt’s report on ‘The electric treatment of the insane’ (1872; 2:203–22) was later credited by Todd and Ashworth as prefiguring the work of Cerletti, which eventually led to electroconvulsive therapy,^15^ but Allbutt’s ‘electrotherapy’ was not inducing seizures (strangely this paper is not referenced by Allbutt’s biographer,^19^ perhaps because of Allbutt’s implication that, although he originally suggested the work, it was mostly undertaken by Major and other resident officers at WRA).

None of these studies was ‘controlled,’ rather than opportunistic. Some data are presented as averages or percentages, but certainly the modern reader is struck by the total absence of statistical analysis. However, a reasonable first at a crossover trial (admittedly unrandomized and unblinded) of different dietary regimens, farinaceous versus nitrogenous, in patients with epilepsy was attempted by Merson (1875; 5:1–23). Although George and Trimble subsequently characterized this work as ‘based on older thinking’^13^ Merson was in fact quite explicit that the direct stimulus to his study was the current thinking of Hughlings Jackson on the investigation of epilepsy, as detailed in one of his five *WRLAMR* papers (1873; 3:326–8).

Pathological work was both macroscopic and microscopic. Crochley Clapham reported on brain weight in the insane (1873; 3:285–298 and 1876; 6:11–26) and with Henry Clarke, Surgeon to the West Riding Prison, on ‘The cranial outline of the insane and criminal’ (1876; 6:150–69). However, it was the microscopical work of Major and Bevan Lewis that was of particular note. In addition to examining senile atrophy in the human brain, Major also examined ageing dog, horse and cat brains in search of analogous changes. He also described an instrument of his own design, the tephrylometer, to measure the thickness of the grey matter (1872; 2:157–76). Bevan Lewis, appointed at WRA in 1875, had little opportunity to contribute to *WRLAMR*, and it was in *Brain* that many of his most significant works subsequently appeared.^25^

As for experimental animal work, this is the sphere in which *WRLAMR* is most likely to be remembered, principally as a consequence of the work (detailed below) of David Ferrier in the dedicated laboratory at WRA, the facilities of which had been offered to him by Crichton-Browne in 1873. Whilst Ferrier was perhaps the chief beneficiary of these facilities, a reading of *WRLAMR* indicates that he was certainly not alone. Animal experimentation was also described in papers by Burman (1872; 2:1–40) using cats, dogs, rabbits, pigeons, frogs and guinea pigs; Aldridge (1872; 2:223–53) using kittens and rats; Lawson (1874; 4:40–84) using cats, rabbits, dogs, pigeons and guinea pigs; Benham (1874; 4:152–78 and 1874; 4:305–17) using dogs, rabbits, pigeons and frogs; Lauder Brunton (1874; 4:179–222) using kittens; and both Arbuckle (1875; 5:130–48) and Bevan Lewis (1876; 6:43–64) using rabbits. Burman reported ‘great difficulty in getting dogs’ whereas ‘a good supply of [rabbits] one can always procure’ (1872; 2:10), whilst Aldridge bemoaned the fact that ‘some large rats were the only animals I could obtain’ (1872; 2:241). Nowhere do mice appear as experimental subjects. Ferrier’s cerebral localization studies on monkeys, although commenced in 1873 [he reported ‘I have now (June 14) ascertained the position of all these [cerebral] centres in the brain of the monkey, and therefore, by implication, their situation in man. These experiments will soon be published.’ 1873; 3:89 footnote 2], were undertaken in London with funding from the Royal Society.^26^ Nevertheless, apes, or at least their brain tissues, were available at the WRA laboratory since Herbert Major’s 1875 thesis, *Histology of the brain in apes*, was based on work done at Wakefield. Some human experimentation also occurred, for example Mitchell inhaling nitrous oxide (1871; 1:44), and Burman injecting himself and other medical officers (Courtenay, Mitchell) with conia (1872; 2:1 422).

The details of the experimental animal work can make for harrowing reading and are reflective of contemporary practices, which provoked and energized the anti-vivisectionist movement. Ferrier was later (1881) to be targeted (unsuccessfully) for attempted legal sanction for his experimental work on monkeys under the 1876 Cruelty to Animals Act,^27^ but this did not relate to his work at WRA. Evidently, however, he was alive to the possibilities of criticism even in 1873: in his first (and seminal) *WRLAMR* paper, based on studies of pigeons, fowls, guinea pigs, rabbits, cats and dogs, he ‘mentioned here, once for all, that before and throughout all the following experiments, ether or chloroform was administered’ (1873; 3:35).^26^

### Contributors and contributions

Finn reported that ‘fifty-eight of the articles came from officers or clerks of the asylum … whilst the remaining twenty-two came from outside contributors, seven of which were based on research conducted in Wakefield’ but gives no further breakdown.^2^ As a ‘house journal’, this preponderance of papers of local origin is to be anticipated. For example, following Crichton-Browne’s inaugural paper, the next two articles were authored by Samuel Mitchell and George Thomson, both AMOs appointed in 1867 following the resignation of the two incumbents and hence both Crichton-Browne’s men. Many of the in-house studies were undoubtedly triggered by suggestions from Crichton-Browne, at times openly acknowledged (e.g. Benham 1874; 4:152; Lawson 1874; 4:243). Crichton-Browne (1876; 6:173) cites the ‘valuable investigations into particular aspects of general paralysis’ by Allbutt, Aldridge, Thompson, Milner Fothergill, Major, Merson, Burman and Newcombe.

To be successful, a research programme generally needs to involve a community of experts, and one of Crichton-Browne’s abilities was to persuade established clinicians to work at WRA and/or to publish in *WRLAMR*. Clifford Allbutt was an early contributor (1872; 2:203–22), as was William Browne (1872; 2:278–301). Annual meetings, or *conversazione*, held at WRA were an opportunity to invite distinguished speakers and the two ‘Papers read at West Riding’,^11^ which appeared in *WRLAMR* were given at these meetings, by the anatomist William Turner (1873; 3:1–29) and the physiologist William Carpenter (1874; 4:1–23). Other external authorities who visited WRA to see patients and/or undertake experimental work included the otolaryngologist Lennox Browne and the physicians Lauder Brunton (1844–1916) and Milner Fothergill. It may be no coincidence that Ferrier, on first moving to London in the early 1870s, shared a house with the latter two^28^; all three, like Crichton-Browne, were Edinburgh medical graduates. However, the most notable contributors to *WRLAMR*, who significantly raised the profile of the journal, were David Ferrier (three papers) and John Hughlings Jackson (five papers), both based in London.

According to Sherrington’s obituary of Ferrier,

‘In March 1873, when he [Ferrier] was paying a visit to his friend … James Crichton-Browne … conversation turned upon the excitability under galvanism of part of the cerebral surface of the dog reported by … Fritsch and Hitzig [1870]. There followed, during the course of the spring and summer of 1873, in the laboratory recently founded at the Asylum … the memorable experiments with which Ferrier opened his detailed exploration by faradic stimulation of all parts of the central nervous system in representative types of vertebrate from the lowest to the highest’.^28^

Ferrier’s work in the WRA laboratory obviously prospered, since as early as 26 April 1873 he published a preliminary note in the *British Medical Journal*,^29^ and subsequently in the *Journal of Anatomy and Physiology*,^30^ both bearing the same title as the substantive work that appeared in *WRLAMR* (1873; 3:30–96). At 67 pages it is the longest paper in the six volumes of *WRLAMR*, and possibly the most significant, in terms of developing both the concept and understanding of cortical localization. In his second *WRLAMR* paper (1874; 4:30–62), Ferrier extended his experimental work to the clinical domain, attempting clinicopathological correlation in five patients from details in the WRA case books ‘to show the clinical bearings of experimental researches on the functions of the brain’. This paper cited Roberts Bartholow’s (1831–1904) now infamous study of cerebral stimulation in a human patient (Mary Rafferty, who had a cancerous ulceration causing a hole in her skull and died a few days after the experiment) published in April 1874,^31,32^ and also mentioned the American ‘crowbar case’, evidently Phineas Gage, but without citation.

Ferrier saw his experimental work as testing and confirming the clinical inferences made by Hughlings Jackson, particularly of epilepsy as a consequence of discharging lesions in the cerebral hemispheres.^33^ Four of Jackson’s five papers in *WRLAMR* related to epilepsy, including observations on cerebral localization of movements based, *inter alia*, on patients with seizures (1873; 3:175–95), the investigation of epilepsies by anatomical, physiological and pathological means (1873; 3:315–49), the temporary mental disorders after epileptic paroxysms (1875; 5:105–29), and the after effects of epileptic discharges (1876; 6:266–309) such as the paresis named after Robert Bentley Todd (1809–60). Samuel Greenblatt has defined the second of these papers as a turning point, as for the first time Jackson focussed on a single subject in a way that his previous papers, invoking evidence from various clinical subjects, had not.^33^ George and Trimble opined that ‘By far the most important writings in the *WRLAMR* are those by J Hughlings Jackson’.^13^

Attempts to understand clinical phenomena in light of cortical localization were also undertaken by other authors, for example Crichton-Browne in his reports on cranial injury (1872; 2:97–136) and the pathology of general paralysis (1876; 6:170–231) (wherein he followed Ferrier’s error of attributing visual functions to the angular gyrus). William Browne summarized some of the emerging literature on aphasia as a consequence of focal brain lesions (1872; 2:278–301). Ferrier’s findings on cerebral localization were subsequently discussed by Carpenter in his address to the WRA *conversazione* of November 1873 (1874; 4:1–23).

The vast majority of contributions to *WRLAMR* were single author papers with only occasional exceptions (namely Nicol and Dove, 1871; 1:233–51; Clapham and Clarke, 1876; 6:150–69; Medical Officers of the West Riding Asylum, 1876; 6:232–51. Lawson and Bevan Lewis, 1876; 6:120–49, hardly counts since many of the eight separate sections contain first person singular pronouns). Internal evidence suggests that there was dialogue, cooperation and a sharing of knowledge between the different researchers working at WRA. For example, Ferrier thanked Crichton-Browne, Milner Fothergill, Galton and McDowall for ‘observing and recording results of experiments’ and was ‘indebted to Mr Galton for original sketches and subsequent drawings on the wood of the illustrations [11 in all] which accompany this paper’ (1873; 3:96). Such dialogue between researchers may not have been the case at WRA after Crichton-Browne’s departure.^34^

When referring to ‘contributors’ we may automatically think of the authors of papers, and consequently overlook the actual, yet involuntary, contributors to many of these publications: the patients. Crichton-Browne’s prefatory reference to ‘the mass of interesting material’ concealed the flesh-and-blood lives of those cared for at WRA, some resident for decades and without hope of clinical recovery. In many of the papers in *WRLAMR* patient details appear, such as initials, age and date of admission, sometimes occupation and place of origin, and occasionally photographs, all of which would permit identification by reference to the original asylum case books (now held at West Yorkshire Archive Service; www.wyjs.org.uk). Some of the life stories of WRA patients, all too often overlooked, have been investigated by these means, both at Wakefield^35,36^ (some for mostly earlier^37^ or later periods^34^) and at Menston (for a later period^38^).

### *WRLAMR* ends, *Brain* begins

**Figure 1:**
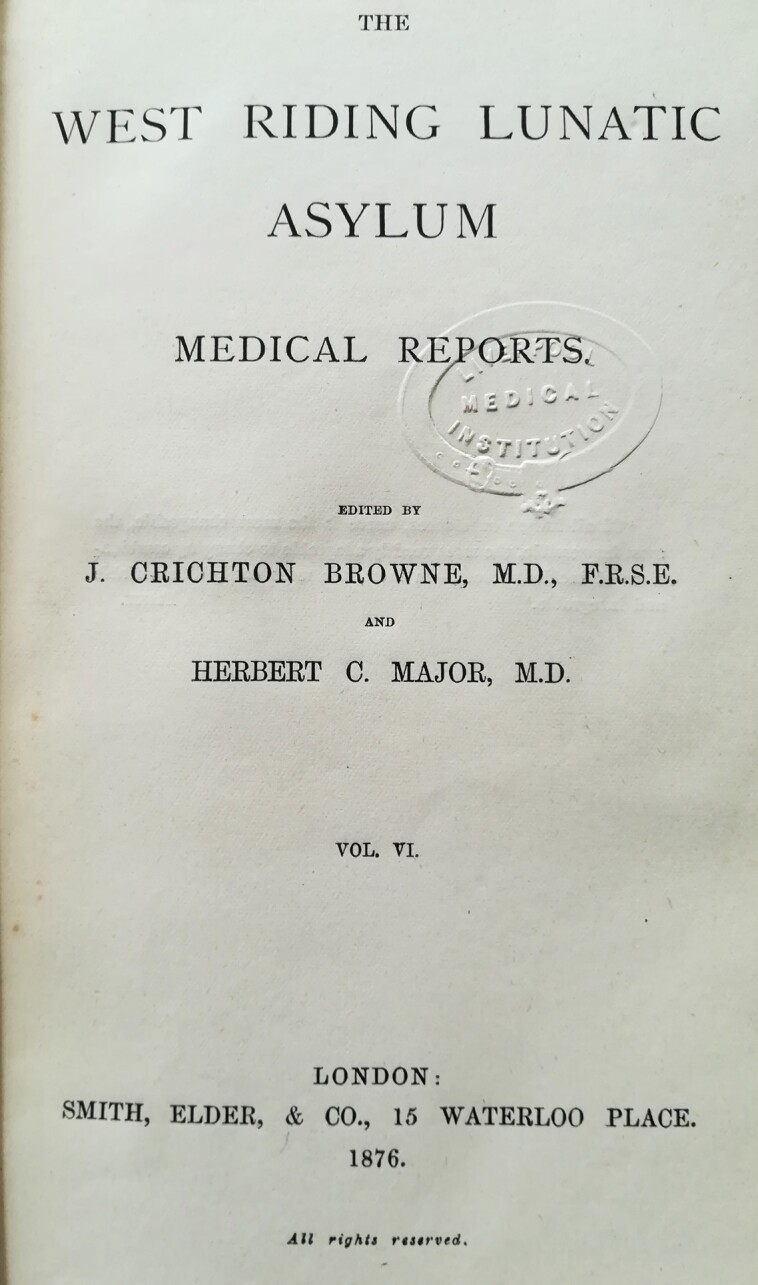
**Title page of the sixth and final volume of the *West Riding Lunatic Asylum Medical Reports*.** Despite the publication date of 1876, internal evidence suggests a *terminus post quem* (a date after which) of early 1877, just over a year before the first issue of *Brain* appeared in April 1878.

Only six volumes of *WRLAMR* were published. The final volume (Fig. 1) appeared after Crichton-Browne’s resignation and departure from WRA and was co-edited by Herbert Major, the new superintendent. Secondary sources state that Major was not willing to continue with the journal,^20,39^ and certainly the unsigned preface to the final volume (which I take to be the work of Major) consists of a single, perfunctory sentence. Although the precise reasons for discontinuation are unknown, it is possible that the ever-growing patient population at WRA, the burden of administrative duties, and a failure to recruit suitable AMOs and Clinical Assistants, all may have contributed to Major’s decision. It was certainly in keeping with the Lunacy Commissioners policy of discouraging superintendents from undertaking research that might distract them from administrative duties. Another possibility is that Crichton-Browne intended the journal to end with his departure. According to Oppenheim, Crichton-Browne had ‘persuaded Churchill, the London medical publisher, to bring out the first two volumes and lined up Smith, Elder for the last four’,^16^ a wording which might suggest that he foresaw only six volumes. If so, Major may justifiably have felt no obligation to continue. However, research efforts did continue at WRA after Crichton-Browne’s departure,^34^ particularly under the long superintendency of Bevan Lewis (1884–1910).^25^

That *WRLAMR* had fulfilled a need, had found a niche, such that its demise left a gap in the market, is perhaps evidenced by the prompt appearance of another journal devoted to similar topics: *Brain: A Journal of Neurology*. The origins of *Brain* are, regrettably, obscure: no records of its founding and operations are apparently extant for its first 25 years (according to a personal communication to Samuel Greenblatt from Professor Alastair Compston, then Editor of *Brain*, dated October 2011).^33^ However, Sherrington, in his obituary of Ferrier, stated that:

‘On the suspension of the West Riding Medical Reports [*sic*], writes Sir James Crichton-Browne, referring to the changes consequent on his own retirement from Wakefield, it was Ferrier who urged that the work which the Reports had begun, should be in some form continued. It then came to be agreed that a “Neurological Journal” should be started in London, and thus “Brain” was launched in 1879 [*sic*]’.^28^

Written fully 50 years after the event, it is not clear what credence may be accorded to this account, doubts possibly compounded by Sherrington’s error in the launch date of *Brain*.

The title page of the first issue of the first volume of *Brain*, dated April 1878, listed the editors (in alphabetical order) as J. C. Bucknill, J. Crichton-Browne (*sic*, with hyphen), D. Ferrier and J. Hughlings-Jackson (*sic*, with hyphen). Three members of this quadrumvirate were, as already discussed, closely associated with *WRLAMR*, the exception being John Charles Bucknill (1817–97). Although Bucknill did not publish in *WRLAMR*, he lectured at WRA, at the annual meeting (*conversazione*) of November 1874. Furthermore, he had prior editorial experience, having been at the helm of the *Asylum Journal*, later the *Journal of Mental Science* (and even later the *British Journal of Psychiatry*), from its inception in 1853 until 1862 when he was appointed Chancery Visitor in Lunacy (the post taken up by Crichton-Browne on his departure from WRA in 1876). There is, to my knowledge, no evidence to support the assertion that Crichton-Browne edited *WRLAMR* ‘with the assistance’ of Jackson, Ferrier and Bucknill and that ‘Sir James encouraged the editorial group to remain with him and founded the present-day journal *Brain*’.^40^

*Brain* was initially published in London by Macmillan and Co., in quarterly instalments appearing in April, July, October and January, such that each volume overlapped 2 years. Issues varied only slightly in length, all around 145 pages. Hence the total pagination for each of the early volumes of *Brain* was approximately twice that of *WRLAMR*. The extra space permitted the contents to be presented under a number of subheadings, namely: Original articles; Critical Digests and Notices of Books; Clinical Cases; and Abstracts of British and Foreign Journals. Unlike *WRLAMR*, the first issue of *Brain* had no editorial preface, but launched directly into the first paper, Jonathan Hutchinson’s ‘Notes on the symptom-significance of different states of the pupil’ [*Brain*. 1878; 1(1):1–13]. However, an anonymous ‘News’ item in the April 1878 issue of the journal *Mind* (1878; 3:295) might serve as *Brain’s* editorial manifesto:

‘The Journal will … include in its scope all that relates to the anatomy, physiology, pathology and therapeutics of the Nervous System. The functions and diseases of the nervous system will be discussed both in their physiological and psychological aspects; but mental phenomena will be treated only in correlation with their anatomical substrata, and mental disease will be investigated as far as possible by the methods applicable to nervous diseases in general’.

### *WRLAMR* compared with *Brain*: 1878–9 to 1883–4

For the purposes of further comparison with *WRLAMR*, the first six volumes of *Brain* (i.e. 1878–9 to 1883–4) are considered. This limitation not only matches the number of volumes of *WRLAMR* but also, conveniently, extends to the time when Armand de Watteville (1846–1925) took over from the quadrumvirate to become the ‘first single editor’ or the ‘acting editor’ of *Brain* (1884).^20^ Furthermore, it has been suggested that after the sixth volume of *Brain*, Crichton-Browne contributed no further original articles.^16^

Aside from Crichton-Browne, many of the authors who had contributed to *WRLAMR* are also to be found in first six volumes of *Brain*, albeit that many were no longer working at or associated with WRA (Supplementary Table 2). However, aside from Hughlings Jackson, Ferrier and Allbutt, none of these contributors may now be considered a familiar name in the neurological world, with the possible exception of Robert Lawson whose description of alcoholic amnesia predated the publication on this subject by Korsakoff.^41^ No less than four *WRLAMR* contributors published in the first issue of *Brain* in April 1878: Allbutt (pp. 60–78), Bevan Lewis (pp. 79–96), Crochley Clapham (pp. 97–100; a subsequent editor of *Brain* was to label this work on skull mapping as ‘verging on phrenology’^42^), and Ferrier (pp. 101–8), these contributions accounting for about one-third of the page content of the first issue. Several other *WRLAMR* contributors appeared in first volume of *Brain* (1878–9): Lauder Brunton, Clarke, Crichton-Browne (who had permission to access WRA records for his later publications^2^), Milner Fothergill, J.C. Galton, Hughlings Jackson, Lawson, Rabagliati and Sankey, a total of 13 in all [this number differs from that reported by Todd and Ashworth who state that ‘nine of the contributors to the first volume (of *Brain*) had had papers published in the *Medical Reports*’^15^ but they give no further details which might permit clarification of this discrepancy]. A 14th *WRLAMR* author, Charles Newcombe, appeared in the second volume of *Brain*. The former *WRLAMR* authors making the most extensive contributions to *Brain* in its first 6 years were Ferrier, Hughlings Jackson and Bevan Lewis (Supplementary Table 2).

From the outset, *Brain* had an international dimension. Not only did it contain ‘Abstracts of British and Foreign Journals’ but also published contributions from authors working outside the UK. In the first issue, H. Duret from the Faculté de Médecine, Paris, was a contributor [1878–9; 1(1):29–47]. In the first six volumes there were not only further papers from France (by Magnan, Foville and Grasset) but also contributions from Austria (Obersteiner, Krueg), USA (Weir Mitchell, Charles K. Mills, Horatio Bigelow, Isaac Ott, Austin Flint, David Yandell), and Germany (Erb, Westphal). No author in *WRLAMR* was from outside the UK, although William Carpenter, perhaps wishing to emphasize his international credentials, chose to give as his affiliation as ‘Corresponding Member of the Institute of France’ (1874; 4:1). If de Watteville, of Swiss/French origin, was assisting *Brain*’s editorial quadrumvirate as early as 1880, as suggested by the Critchleys,^39^ this may also have contributed to its international perspective.^12^

In addition to their contributions to *Brain*, it may be noted that several *WRLAMR* authors made further contributions to contemporary neuroscience in various other ways. For example, J.C. Galton translated from the German Alexander Ecker’s *On the convolutions of the human brain* (1873), a work used by Ferrier to transpose his maps of cerebral localization in the monkey cortex on to diagrams of the human cerebral convolutions. Galton’s translation, like Ferrier’s monograph^43^ and the final four volumes of *WRLAMR*, was published by Smith, Elder, & Co. A patient of Milner Fothergill’s, referred to Hughlings Jackson, was reported by Jonathan Hutchinson in the second instalment of his ‘Notes on the symptom-significance of different states of the pupil’, which appeared in the second issue of *Brain* [1878; 1(2):155–67]. Bevan Lewis, in addition to his own extensive pathological publications in *Brain* (Supplementary Table 2), contributed the microscopical examination of Thomas Buzzard’s patient with ophthalmoplegia externa in *tabes dorsalis* [*Brain* 1882; 5(1):34–40]. With Henry Clarke, Bevan Lewis published a seminal paper on cytoarchitectonics, building on the work of Meynert and illustrating for the first time the large pyramidal cells originally described by Betz.^44^ Octavius Sankey later (1877) co-authored a paper on chorea with W.R. Gowers.^45^

## Discussion

*WRLAMR* is a defunct journal of the 19th century. As such, many neurologists are unlikely to be aware of it and its relationship to *Brain*. Various opinions have been expressed regarding this relationship. For example, Oppenheim stated that ‘the earlier, little known journal paved the way for the later, famous one’^16^ and George and Trimble also describe *WRLAMR* as ‘little-known’.^13^ Whilst it may be accepted that *WRLAMR* is little known to posterity, contemporary evidence suggests it was in fact well known in the field, with reviews appearing in many journals including, but not limited to, *The Lancet*, *British Medical Journal*, *Journal of Mental Science*, *Boston Medical and Surgical Journal* (forerunner of the *New England Journal of Medicine*), *American Journal of Insanity* (forerunner of the *American Journal of Psychiatry*), and the *Journal of Nervous and Mental Diseases*. Ferrier’s 1873 *WRLAMR* article also gave the journal considerable profile.^26^

Jellinek’s view was that *Brain* was similar to *WRLAMR* in its range of contents but was more international in outlook.^1^ Certainly the latter view is supported by the current analysis. Finn concluded that ‘The most obvious … legacy of the [West Riding] asylum’s work … was the neurological journal *Brain*, which was essentially a continuation of the asylum’s own *Reports*’.^2^ However, as has been shown, the content of *Brain* was more extensive and diverse than that of *WRLAMR*, in part because of the greater space available.

Undoubtedly there were continuities between *WRLAMR* and *Brain*: as has been shown, many of the significant players in terms of editing and contributing played similar roles in both journals, and there was overlap of subject matter. But there were also important discontinuities, perhaps most clearly manifest in the designation of *Brain* as ‘a journal of neurology’ thus clearly distinguishing it from the constituency of asylum clinicians, which *WRLAMR* had set out to serve, a point also emphasized in the ‘News’ item on the new journal, which appeared in *Mind*. If Ferrier was, as per Sherrington’s obituary account,^28^ the principal moving force in initiating *Brain* then this emphasis may be easily explained. Thus, the recent view that ‘Ferrier, Crichton-Browne, John Bucknill and John Hughlings Jackson … rebranded *The West Riding Lunatic Asylum Medical Reports* into the journal *Brain*’^46^ does not appear consistent with the evidence.

Over 150 years since the first publication of *WRLAMR*, it may be possible to assess to what extent Crichton-Browne’s prefatory hopes that ‘the series may in some measure conduce to the relief of suffering, the advancement of science, and the credit of the medical profession’ (1871; 1:v) were fulfilled. The relief of suffering is extremely doubtful, unless viewed indirectly, since none of the treatments examined and advocated is still in clinical use. As to the advancement of science, this certainly was fulfilled, both experimentally by Ferrier and clinically by Hughlings Jackson: their ideas regarding cortical localization remain central to neurological practice and neuroscientific research. By this same token one might argue that, as Crichton-Browne hoped, *WRLAMR* contributed to the credit of the medical profession.

For these reasons, I submit that it is legitimate to conclude that *WRLAMR* was not only a precursor of *Brain*, as has previously been suggested,^12^ but in actuality a precondition for the emergence of *Brain*. Hence, as E.D. Adrian opined, the endeavours of those working at WRA and manifested in the pages of *WRLAMR* indeed link us to ‘a classical period in the history of medicine, the period when neurology became a science’.^47^

## Supplementary Material

awad219_Supplementary_Data
