# Supplementary material for: The West Riding Lunatic Asylum Medical Reports: the precursor of Brain?
Source: Brain. 2023 Jul 3;146(11):4437–45. doi: 10.1093/brain/awad219 (PMC10805577; doi:10.1093/brain/awad219)
Supplement: awad219_Supplementary_Data [file awad219_supplementary_data.zip › brain-2023-00244-File003.pdf]

Supplementary Table S1: *WRLAMR* contents by volume

NB The titles of some articles differ between the tables of Contents and the title page of the paper.

Volume 1 (1871)
Available online at:
https://wellcomecollection.org/works/v3kfekjk/items
http://dbooks.bodleian.ox.ac.uk/books/PDFs/502369335.pdf
https://archive.org/details/39002086346260.med.yale.edu

**Reference**	**Title**	**Author and qualifications**	**Affiliation(s)**
I: i	[Title page]		
I: ii	[Epigraph]	Berkeley	
I: iii-v	Preface	[JCB]	
I: vi	[blank]		
I: vii-viii	Contents		
I: 1-26 [1]	Cranial injuries and mental diseases	James Crichton Browne [*sic*, without hyphen] MD FRSE	Medical Director West Riding Asylum, and Lecturer on Mental Diseases to the Leeds School of Medicine
I: 27-57 [2]	Observations on the physiological action of nitrous oxide	Samuel Mitchell MD	Deputy Medical Director West Riding Asylum, and Physician to the Sheffield Hospital and Dispensary
I: 58-70 [3]	The sphygmograph in lunatic asylum practice	George Thompson LRCP Lond	Medical Superintendent of the Bristol City and County Lunatic-Asylum; late Assistant Medical Officer, and formerly Clinical Assistant, at the West Riding Asylum, Wakefield
I: 71-128 [4]	The opthalmoscope [*sic*; cf. "Contents"] in mental and cerebral diseases	Charles Aldridge LRCP Lond	Assistant Medical Officer, Late Clinical Assistant, West Riding Asylum, Wakefield
I: 129-151 [5]	A contribution to the statistics of general paralysis; with remarks	J Wilkie Burman MB (Edin)	Assistant Medical Officer, West Riding Asylum; Late Assistant Medical Officer of the Devon County Asylum, and Clinical Assistant, West Riding Asylum
I: 152-163 [6]	On the treatment of insanity by the hypodermic injection of morphia	J Bywater Ward BA MB Cantab	Assistant Medical Officer Warwick County Asylum; Late Clinical Assistant, West Riding Asylum
I: 164-177 [7]	Mollities ossium and allied diseases	George Henry Pedler LRCP Lond, MRCS	Fellow of the Obstetrical Society, and Late Clinical Assistant, West Riding Asylum
I: 178-208	On progressive	Patrick Nicol	Physician to the Bradford

[8]	locomotor ataxy and some other forms of locomotor deficiency as found in the insane	MA MB	Infirmary, Late Assistant Medical Officer, Sussex County Asylum, and Clinical Assistant, West Riding Asylum
I: 209-217 [9]	On the artificial feeding of the insane	William Lawrence MB	Assistant Medical Officer Chester County Asylum; Late Clinical Assistant West Riding Asylum
I: 218-232 [10]	Arachnoid cysts	Henry Sutherland MA MB Oxon MRCP Lond	Physician to the St George's, Hanover Square, Dispensary, and Late Assistant Medical Officer, West Riding Asylum
I: 233-251 [11]	Phthisis and insanity	Patrick Nicol MA MB<br><br>W Watson Dove LRCPE MRCS	Physician to the Bradford Infirmary; Late Assistant Medical Officer, Sussex County Asylum; and Clinical Assistant West Riding Asylum<br><br>Assistant Medical Officer, Somerset County Asylum; Late Clinical Assistant, West Riding Asylum
I: 252-260 [12]	Acute delirious melancholia	Charles Henry Mayhew LRCP Lond MRCS	Associate of King's College, Assistant House-Surgeon to the Stockport Infirmary, formerly Clinical Assistant, West Riding Asylum
I: 261-265 [13]	Ergot of rye in the treatment of mental diseases	E Churchill Fox MB CM	Late Clinical Assistant, West Riding Asylum

Volume 2 (1872)
Available online at:
http://dbooks.bodleian.ox.ac.uk/books/PDFs/555022684.pdf
https://archive.org/details/39002086346278.med.yale.edu

Reference	Title	Author	Affiliation(s)
II: i	[Title page with editor and publisher]		
II: ii	[Epigraph]	Emerson; Shakespeare	
II: iii	Preface	[JCB]	
II: iv	[blank]		
II: v-vi	Contents		
II: 1-40 [1]	On conia, and its use in subcutaneous injection	J Wilkie Burman MD (Edin)	Assistant Medical Officer, Male Division, West Riding Asylum; Late Assistant Medical Officer, Devon County Asylum; and Clinical Assistant, West Riding

			Asylum
II: 41-52 [2]	On the minute structure of the cortical substance of the brain, in a case of chronic brain wasting	Herbert C Major MB CM (Edin)	Assistant Medical Officer, Late Clinical Assistant, West Riding Asylum
II: 53-72 [3]	Menstrual irregularities and insanity	Henry Sutherland MA MB Oxon MRCP Lond	Lecturer on Insanity to the Westminster Hospital School of Medicine; Late Assistant Medical Officer, West Riding Asylum
II: 73-96 [4]	Experiments to ascertain the effects of ether and nitrous oxide combined, to which are added some general observations on stimulants	Samuel Mitchell MD	Medical Superintendent, South Yorkshire Asylum, Late Assistant Medical Officer, West Riding Asylum
II: 97-136 [5]	Cranial injuries and mental diseases	James Crichton Browne [*sic*, without hyphen] MD FRSE	Medical Director, West Riding Asylum, and Lecturer on Mental Diseases to the Leeds School of Medicine
II: 137-156 [6]	Puerperal mania	George Henry Pedler LRCP Lond, MRCS	Fellow of the Obstetrical Society, and Late Clinical Assistant, West Riding Asylum
II: 157-176 [7]	A new method of determining the depth of the grey matter of the cerebral convolutions	Herbert C Major MB	Assistant Medical Officer, Late Clinical Assistant, West Riding Asylum
II: 177-202 [8]	The mental symptoms of ordinary disease	Patrick Nicol MA MB	Physician to the Bradford Infirmary, Formerly Clinical Assistant, West Riding Asylum
II: 203-222 [9]	The electric treatment of the insane	T Clifford Allbutt MA MD (Cantab) FLS	Physician to the Leeds General Infirmary, &c.
II: 223-253 [10]	Ophthalmoscopic observations in general paralysis, and after the administration of certain toxic agents	Charles Aldridge LRCP Lond	Assistant Medical Officer, Late Clinical Assistant, West Riding Asylum
II: 254-277 [11]	The use of opium in the treatment of melancholia	E Maziere Courtenay MB AB TCD	Assistant Medical Officer, Derby County Asylum, Late Clinical Assistant, West Riding Asylum
II: 278-	Impairment of	W A F Browne	Psychological Consultant,

301 [12]	language, the result of cerebral disease	MD FRCSE FRSE	Crichton Royal Institution, Late Commissioner in Lunacy for Scotland
II: 302-306 [13]	The sphygmograph in epilepsy	George Thompson LRCP Lond	Medical Superintendent, Bristol County and City Lunatic-Asylum; Late Assistant Medical Officer and Clinical Clerk, West Riding Asylum

Volume 3 (1873)
Available online at:
https://archive.org/details/39002086346286.med.yale.edu

**Reference**	**Title**	**Author**	**Affiliation(s)**
III: i	[Title page with editor and publisher]		
III: ii	[Epigraph]	Carlyle	
III: iii-iv	Preface	[JCB]	
III: v-vi	Contents		
III: 1-29 [1]	The convolutions of the human brain considered in relation to the intelligence	William Turner MB Lond	Professor of Anatomy in the University of Edinburgh
III: 30-96 [2]	Experimental researches in cerebral physiology and pathology	David Ferrier MA MD (Edin) MRCP	Professor of Forensic Medicine, King's College London; Assistant Physician to the West London Hospital
III: 97-112 [3]	Observations on the histology of the brain in the insane	Herbert C Major MB CM	Assistant Medical Officer, Late Clinical Assistant, West Riding Asylum
III: 113-128 [4]	The heart sounds in general paralysis of the insane	J Milner Fothergill MD MRCP	[No affiliation given]
III: 129-152 [5]	On the power of perceiving colours possessed by the insane	T W McDowall MD	Pathologist, and Assistant Medical Officer, West Riding Asylum
III: 153-174 [6]	Nitrite of amyl in epilepsy	James Crichton Browne [*sic*, without hyphen] MD (Edin) FRSE	Medical Director, West Riding Asylum, and Lecturer of Mental Diseases to the Leeds School of Medicine
III: 175-195 [7]	Observations on localisation of movements in the cerebral hemispheres, as revealed by cases of convulsion, chorea and 'aphasia'	J Hughlings Jackson MD FRCP	Physician to the London Hospital and to the Hospital for the Epileptic and Paralysed

III: 196-215 [8]	On electro-excitability in mental and nervous diseases	John Lowe MB CM (Edin)	Assistant Medical Officer, South Yorkshire Asylum; Late Assistant Medical Officer, Durham County Asylum; and Clinical Assistant, West Riding Asylum
III: 216-257 [9]	Heart disease and insanity	J Wilkie Burman MD (Edin)	Deputy Medical Director, West Riding Asylum, Wakefield
III: 258-272 [10]	Notes on the condition of the tympanic membrane in the insane – Part I	John C Galton MA (Oxon) MRCS FLS	Clinical Assistant, West Riding Asylum
III: 273-284 [11]	On the obscurer neuroses of syphilis	T Clifford Allbutt MA MD (Cantab) FLS	Physician to the Leeds General Infirmary
III: 285-298 [12]	The weight of the brain in the insane	W Crochley S Clapham Esq. LRCP (Lond)	Fellow of the London Anthropological Society; Late Clinical Assistant West Riding Asylum
III: 299-314 [13]	The change of life, and insanity	Henry Sutherland MD MA (Oxon) MRCP (Lond)	Lecturer on Insanity to the Westminster Hospital; Late Assistant Medical Officer West Riding Asylum; Physician to the St. George's Hanover Square Dispensary; Etc.
III: 315-349 [14]	On the anatomical, physiological, and pathological investigation of epilepsies	J Hughlings Jackson MD FRCP	Physician to the London Hospital, and to the Hospital for the Epileptic and Paralysed

Volume 4 (1874)
Available online at:
https://archive.org/details/39002086346294.med.yale.edu

Reference	Title	Author	Affiliation(s)
IV: i	[Title page]		
IV: ii	[blank]		
IV: iii	[Title page with editor and publisher]		
IV: iv	[Epigraph]	Swedenborg	
IV: v	Preface	[JCB]	
IV: vi	[blank]		
IV: vii-viii	Contents		
IV: 1-23	On the	William B Carpenter	Corresponding Member of the

[1]	physiological import of Dr Ferrier's experimental investigations into the functions of the brain	MD LLD FRS	Institute of France
IV: 24-29 [2]	On a case of recovery from double optic neuritis	J Hughlings Jackson MD FRCP	Physician to the London Hospital, and to the Hospital for the Epileptic and Paralysed
IV: 30-62 [3]	Pathological illustrations of brain function	David Ferrier MD MRCP	Professor of Forensic Medicine, King's College, London; Assistant-Physician to King's College, London.
IV: 63-93 [4]	The urinology of general paralysis	John Merson MA MD	Assistant Medical Officer, West Riding Lunatic Asylum
IV: 94-151 [5]	Cerebral anaemia	J Milner Fothergill MD Edin MRCP Lond	[No affiliation given]
IV: 152-178 [6]	On the therapeutic value of cold to the head	William T Benham MD Abdn	Pathologist and Assistant Medical Officer, West Riding Asylum
IV: 179-222 [7]	On inhibition, peripheral and central	T Lauder Brunton MD DSc Edin FRS MRCP	Casualty Physician and Lecturer on Materia Medica and Therapeutics at St Bartholomew's Hospital
IV: 223-239 [8]	Observations on the histology of the morbid brain	Herbert C Major MB Edin	Deputy Medical Director, West Riding Asylum
IV: 240-264 [9]	On the hourly distribution of mortality in relation to recurrent changes in the activity of vital functions	Robert Lawson MB CM Edin	Clinical Assistant, West Riding Asylum; Formerly Assistant to the Professor of Practice of Medicine and Medical Psychology at Edinburgh University
IV: 265-290 [10]	Acute dementia [cf. Contents: On acute dementia]	James Crichton Browne MD FRSE	Medical Director, West Riding Asylum; and Lecturer on Mental Diseases to the Leeds School of Medicine
IV: 291-304 [11]	Ophthalmoscopic observations in acute dementia	Charles Aldridge MB	Physician to the Plimpton House Asylum; late Senior Assistant Medical Office; formerly Clinical Assistant, West Riding Asylum, Wakefield
IV: 305-317 [12]	The actions of nicotine [cf. Contents: On the actions of	William T Benham MD Abdn	Pathologist and Assistant Medical Officer, West Riding Asylum

	nicotine]		

Volume 5 (1875)
Available online at:
https://archive.org/details/39002086346302.med.yale.edu

Reference	Title	Author	Affiliation(s)
V: i	[Title page]		
V: ii	[blank]		
V: iii	[Title page with editor and publisher]		
V: iv	[Epigraph]	[GH] Lewes	
V: v	Preface	[JCB]	
V: vi	[blank]		
V: vii-viii	Contents		
V: 1-23 [1]	On the influence of diet in epilepsy	John Merson MA MD	Assistant Medical Officer, West Riding Asylum, Wakefield
V: 24-39 [2]	Labyrinthine vertigo. Menière's disease	David Ferrier MD	Assistant Physician to King's College Hospital. Professor of Forensic Medicine, King's College, London
V: 40-84 [3]	On the physiological action of hyoscyamine	Robert Lawson MB Edin	Pathologist and Assistant Medical Officer, West Riding Asylum
V: 85-104 [4]	On the histology of the great sciatic nerve in general paralysis of the insane	W Bevan Lewis LRCP Lond, MRCS, FRMS	Clinical Assistant, West Riding Asylum; Formerly Assistant Medical Officer, Bucks County Asylum
V: 105-129 [5]	On temporary mental disorders after epileptic paroxysms	J Hughlings Jackson MD FRCP	Physician to the London Hospital and to the Hospital for the Epileptic and Paralysed
V: 130-148 [6]	On the appearance of the retina and choroid during the administration of certain drugs	John Hunter Arbuckle MD and CM Glas	Clinical Assistant, West Riding Asylum
V: 149-159 [7]	Othaematoma, or the insane ear	Lennox Browne FRCS Edin	Senior Surgeon to the Central London Throat and Ear Hospital, Surgeon, and Aural Surgeon to the Royal Society of Musicians, etc
V: 160-170 [8]	On the morbid histology of the	Herbert C Major MD Edin	Deputy Medical Director, West Riding Asylum, Wakefield

	brain in the lower animals		
V: 171-187 [9]	Cerebral hyperaemia	J Milner Fothergill MD Edin MRCP Lond	Junior Physician to the West London Hospital
V: 188-197 [10]	A new process for examining the structure of the brain. With a review of some points in the histology of the cerebellum	H R Octavius Sankey	Undergraduate in Medicine of the University of London
V: 198-226 [11]	Epileptiform seizures in general paralysis	Charles F Newcombe MB	Assistant Medical Officer, Lancaster County Asylum, Rainhill. Late Clinical Assistant, West Riding Asylum
V: 227-256 [12]	The functions of the thalami optici	James Crichton Browne MD FRSE	Medical Director, West Riding Asylum
V: 257-270 [13]	On the therapeutic value of chloral hydrate in epileptic convulsions	J A M Wallis [cf. Contents: "A M Wallis"] MD	Medical Superintendent, Hull Borough Asylum. Late Assistant Medical Officer, West Riding Asylum
V: 271-283 [14]	Laryngoscopic observations in general paralysis	Lennox Browne FRCS Edin	Senior Surgeon to the Central London Throat and Ear Hospital, Surgeon to the Royal Society of Musicians, to Her Majesty's Italian Opera, etc
V: 284-292 [15]	Note on chronic mania	James Crichton Browne MD FRSE	Medical Director, West Riding Asylum

Volume 6 (1876)
Available online at:
https://archive.org/details/39002086346310.med.yale.edu

Reference	Title	Author	Affiliation(s)
VI: i	[Title page]		
VI: ii	[blank]		
VI: iii	[Title page with editor and publisher]		
VI: iv	[Epigraph]	Fiske	
VI: v	Preface	[HCM?]	
VI: vi	[blank]		
VI: vii-viii	Contents		
VI: 1-10	The histology of	Herbert C Major	Medical Director West Riding

[1]	the island of Reil	MD	Asylum
VI: 11-26<br>[2]	The weight of the brain in the insane	Crochley Clapham<br>LRCP Lond etc.	West Riding Asylum
VI: 27-42<br>[3]	On classification and nomenclature in nervous disorders	A H Rabagliati<br>MA MD	Bradford
VI: 43-64<br>[4]	Calorimetric observations upon the influence of various alkaloids on the generation of animal heat	W Bevan Lewis<br>LRCP Lond, MRCS	Pathologist and Assistant Medical Officer, West Riding Asylum
VI: 65-84<br>[5]	Hyoscyamine in the treatment of some diseases of the insane	Robert Lawson<br>MB	Assistant Medical Officer, West Riding Asylum
VI: 85-107<br>[6]	The climacteric period in relation to insanity	John Merson<br>MD	Assistant Medical Officer, West Riding Asylum
VI: 108-119<br>[7]	Cases on the borderland of insanity	Henry Sutherland<br>MD MRCP	Lecturer on Insanity to the Westminster Hospital School of Medicine
VI: 120-149<br>[8]	Clinical notes on conditions incidental to insanity	Robert Lawson<br>MB<br><br>W Bevan Lewis<br>LRCP Lond	Assistant Medical Officer, West Riding Asylum<br><br>Pathologist and Assistant Medical Officer, West Riding Asylum
VI: 150-169<br>[9]	The cranial outline of the insane and criminal	Crochley Clapham<br>LRCP Lond<br><br>Henry Clarke<br>LRCP Lond	West Riding Asylum<br><br><br>Surgeon, West Riding Prison
VI: 170-231<br>[10]	Notes on the pathology of general paresis of the insane	James-Crichton Browne<br>[*sic*, with hyphen]<br>MD FRSE	Lord Chancellor's Visitor of Lunatics
VI: 232-251<br>[11]	A case of epilepsy (under the care of Dr Crichton-Browne)	Medical Officers of the West Riding Asylum	-
VI: 252-265<br>[12]	Notes on the therapeutics of some affections of the nervous system	J Milner Fothergill<br>MD MRCP	Assistant Physician to the West London Hospital, etc
VI: 266-309<br>[13]	On epilepsies and on the after effects of	J Hughlings Jackson<br>MD FRCP	[No affiliation given]

	epileptic discharges (Todd and Robertson’s hypothesis)		
