# Supplementary material for: The West Riding Lunatic Asylum Medical Reports: the precursor of Brain?
Source: Brain. 2023 Jul 3;146(11):4437–45. doi: 10.1093/brain/awad219 (PMC10805577; doi:10.1093/brain/awad219)
Supplement: awad219_Supplementary_Data [file awad219_supplementary_data.zip › brain-2023-00244-File004.pdf]

Supplementary Table S2: Authors (listed alphabetically) common to both the six volumes of *WRLAMR* (published 1871 to 1876) and the first six volumes of *Brain* (published 1878-9 to 1883-4)

**Author**	**Publications in *WRLAMR***<br>Title. Year;volume:pages.	**Publications in *Brain***<br>Title. Year;volume(issue):pages.
Allbutt,<br>T Clifford	The electric treatment of the insane. 1872;2:203-22.<br><br>On the obscurer neuroses of syphilis. 1873;3:273-84.	On brain forcing. 1878;1(1):60-78.<br><br>Critical Digests and Notices of Books. 1879;2(1):95-9.<br><br>Critical Digests and Notices of Books. 1879;2(3):385-90.<br><br>Case of epileptiform migrane [*sic*]. 1883;6(2):246-9.
Brunton,<br>T Lauder	On inhibition, peripheral and central. 1874;4:179-222.	Reflex action as a cause of disease and means of cure. 1878;1(2):143-54.<br><br>On the position of the motor centres in the brain in regard to the nutritive and social functions. 1882;4(4):431-40.<br><br>Review and Notices of Books. 1883;6(2):263-6.
Clapham,<br>W Crochley S	The weight of the brain in the insane. 1873;3:285-98.<br><br>The weight of the brain in the insane. 1876;6:11-26.<br><br>The cranial outline of the insane and criminal. 1876;6:150-69. [with H Clarke]	On skull mapping. 1878:1(1):97-100.<br><br>Head measurements. 1880;2(4):591-2.
Clarke,<br>Henry	The cranial outline of the insane and criminal. 1876;6:150-69. [with WCS Clapham]	The effect of seclusion on the body weight. 1878;1(2):210-4.<br><br>Heredity and crime in epileptic criminals. 1880;2(4):491-527.
Crichton-Browne,<br>James	[Preface. 1871;1:iii-v.]<br><br>Cranial injuries and mental diseases. 1871;1:1-26.<br><br>[Preface. 1872;1:iii.]	Critical Digests and Notices of Books. 1878;1(2):215-9.<br><br>Critical Digests and Notices of Books. 1878;1(3):379-82.<br><br>Critical Digests and Notices of

	Cranial injuries and mental diseases. 1872;2:97-136.<br><br>[Preface. 1873;1:iii-iv.]<br><br>Nitrite of amyl in epilepsy. 1873;3:153-74.<br><br>[Preface. 1874;4:v.]<br><br>Acute dementia. 1874;4:265-90.<br><br>[Preface. 1875;5:vi.]<br><br>The functions of the thalami optici. 1875;5:227-56.<br><br>Note on chronic mania. 1875;5:284-92.	Books. 1878;1(3):386-7.<br><br>On the weight of the brain and its component parts in the insane. 1879;1(4):504-18.<br><br>On the weight of the brain and its component parts in the insane. 1879;2(1):42-67.<br><br>Heredity in epilepsy. 1879;2(2):290-1.<br><br>Critical Digests and Notices of Books. 1880;3(1):111.<br><br>Critical Digests and Notices of Books. 1880;3(1):112.<br><br>A plea for the minute study of mania. 1880;3(3):347-62.<br><br>Critical Digests and Notices of Books. 1881;3(4):528-31.<br><br>Critical Digests and Notices of Books. 1881;4(3):392-8.<br><br>Review and Notices of Books. 1883;6(1):120-4.<br><br>The pulmonary pathology of general paralysis. 1883;6(3):317-41.
Ferrier, David	Experimental researches in cerebral physiology and pathology. 1873;3:30-96.<br><br>Pathological illustrations of brain function. 1874;4:30-62.<br><br>Labyrinthine vertigo. Menière's disease. 1875;5:24-39.	Critical Digests and Notices of Books. 1878;1(1):101-8.<br><br>Critical Digests and Notices of Books. 1878;1(2):229-31.<br><br>Critical Digests and Notices of Books. 1878;1(2):239-49.<br><br>Pain in the head in connection with cerebral disease. 1879;1(4):467-83.<br><br>Vomiting in connection with cerebral disease. 1879;2(2):223-33.<br><br>On the termination of nerves in

		striated muscles. 1879;2(2):286-9.<br><br>Critical Digests and Notices of Books. 1879;2(3):400-402<br><br>Critical Digests and Notices of Books. 1880;3(1):85-99.<br><br>Crural monoplegia – limited cortical lesion of opposite hemisphere. 1880;3(1):128-31.<br><br>Tripier on anaesthesia from cortical lesions. 1880;3(2):286-8.<br><br>Critical Digests and Notices of Books. 1880;3(3):365-73.<br><br>Critical Digests and Notices of Books. 1880;3(3):383-95.<br><br>Brachial monoplegia. 1880;3(3):417-27.<br><br>Cerebral amblyopia and hemiopia [*sic*]. 1881;3(4):456-77.<br><br>Critical Digests and Notices of Books. 1881;4(1):111-2.<br><br>Löwenfeld on electro-therapy of the brain. 1881;4(1):136-8.<br><br>The localisation of atrophic paralyses. 1881;4(2):217-32.<br><br>Critical Digests and Notices of Books. 1881;4(2):246.<br><br>Gaucher on the morbid anatomy of diphtheritic paralysis. 1881;4(2):270-6.<br><br>The localisation of atrophic paralyses. 1881;4(3):303-24.<br><br>Straus on tabetic ecchymoses. 1881;4(3):428-30.<br><br>Critical Digests and Notices of

		Books. 1882;4(4):517-8.<br><br>Critical Digests and Notices of Books. 1882;4(4):519.<br><br>Semon on abductor laryngeal paralysis. 1882;4(4):554-62.<br><br>The brain of a criminal lunatic. 1882;5(1):62-73.<br><br>Glioma of the right optic thalamus and corpora quadrigemina. 1882;5(1):123-7.<br><br>Westphal on the knee-phenomenon. 1882;5(2):286-8.<br><br>Case of allochiria. 1882;5(3):389-93.<br><br>Facial monospasm. 1882;5(3):429-32.<br><br>Hemiplegic muscular atrophy of peripheral origin. 1883;5(4):521-8.<br><br>Observations on a case of cerebral cortico-medullary glioma. 1883;6(1):67-77.
Fothergill,<br>J Milner	The heart sounds in general paralysis of the insane. 1873;3:113-28.<br><br>Cerebral anaemia. 1874;4:94-151.<br><br>Cerebral hyperaemia. 1875;5:171-87.<br><br>Notes on the therapeutics of some affections of the nervous system. 1876;6:252-65.	The neurosal [*sic*] and reflex disorders of the heart. 1878;1(2):195-209
Galton,<br>John C	Notes on the condition of the tympanic membrane in the insane – Part I. 1873;3:258-72.	Critical Digests and Notices of Books. 1878;1(3):382-6.
Jackson,<br>John Hughlings	Observations on localisation of movements in the cerebral hemispheres, as revealed by cases of convulsion, chorea and	On affections of speech from disease of the brain. 1878;1(3):304-30.

	"aphasia". 1873;3:175-95.<br><br>On the anatomical, physiological, and pathological investigation of epilepsies. 1873;3:315-49.<br><br>On a case of recovery from double optic neuritis. 1874;4:24-29.<br><br>On temporary mental disorders after epileptic paroxysms. 1875;5:105-29.<br><br>On epilepsies and on the after effects of epileptic discharges (Todd and Robertson's hypothesis). 1876;6:266-309.	Auditory vertigo. 1879;2(1):29-38.<br><br>On affections of speech from disease of the brain. 1879;2(2):203-22.<br><br>Note on Dr. J. Hughlings-Jackson's [*sic*] case of auditory vertigo in April No. of "Brain". 1879;2(2):274.<br><br>On affection of speech from disease of the brain. 1879;2(3):323-56.<br><br>On right or left-sided spasm at the onset of epileptic paroxysms, and on crude sensation warnings, and elaborate mental states. 1880;3(2):192-206.<br><br>Buzzard on certain point in tabes dorsalis. 1880;3(2):266-8.<br><br>On temporary paralysis after epileptiform and epileptic seizures; a contribution to the study of dissolution of the nervous system. 1881;3(4):433-51.<br><br>Buzzard on transfer-phenomena in epilepsy produced by encircling blisters. 1881;3(4):554-5.<br><br>Buzzard on the affection of bones and joints in locomotor ataxy, and its association with gastric crises. 1881;4(2):276-82.<br><br>Localised convulsions from tumour of the brain. 1882;5(3):364-74.<br><br>Critical Digests and Notices of Books. 1882;5(3):382-8.
Lawson,<br>Robert	On the hourly distribution of mortality in relation to recurrent changes in the activity of vital functions. 1874;4:240-64.	On the symptomatology of alcoholic brain disorders. 1878;1(2):182-94<br><br>Hystero-neuroses. 1879;2(1):148.

	On the physiological action of hyoscyamine. 1875;5:40-84.<br><br>Hyoscyamine in the treatment of some diseases of the insane. 1876;6:65-84.<br><br>Clinical notes on conditions incidental to insanity. 1876;6:120-49 [with W Bevan Lewis]	American neurological notes. 1879;2(3):446-8.<br><br>General paralysis amongst negroes. 1880;2(4):592.<br><br>Traumatic insanity. 1880;3(1):143-4.
Lewis,<br>William Bevan	On the histology of the great sciatic nerve in general paralysis of the insane. 1875;5:85-104.<br><br>Calorimetric observations upon the influence of various alkaloids on the generation of animal heat. 1876;6:43-64.<br><br>Clinical notes on conditions incidental to insanity. 1876;6:120-49 [with R Lawson]	On the comparative structure of the cortex cerebri. 1878;1(1):79-96.<br><br>Application of freezing methods to the microscopic examination of the brain. 1878;1(3):348-59.<br><br>In the brain of the insane. 1879;2(3):364-72.<br><br>Methods of preparing, demonstrating, and examining cerebral structure in health and disease. 1880;3(3):314-36.<br><br>Methods of preparing, demonstrating, and examining cerebral structure in health and disease. 1881;3(4):502-15.<br><br>Methods of preparing, demonstrating, and examining cerebral structure in health and disease. 1881;4(1):82-99.<br><br>Critical Digests and Notices of Books. 1881;4(2):238-46.<br><br>Methods of preparing, demonstrating, and examining cerebral structure in health and disease. 1881;4(3):351-60.<br><br>Critical Digests and Notices of Books. 1881;4(3):377-80.<br><br>Methods of preparing, demonstrating, and examining

		cerebral structure in health and disease. 1882;4(4):441-66.<br><br>Histological notes on a case of tabes with ophthalmoplegia externa. 1882;5(1):41-55.<br><br>Methods of preparing, demonstrating, and examining cerebral structure in health and disease. 1882;5(1):74-88.<br><br>Pozzi on sclerosis of the cerebral convolutions. 1883;6(2):285-6.<br><br>On posterior spinal sclerosis, consecutive to disease of blood-vessels: microscopical examinations of the cord [with T Buzzard]. 1884;6(4):461-7.<br><br>On posterior spinal sclerosis, consecutive to disease of blood-vessels: report of microscopical examination of the spinal cord. 1884;6(4):467-80.<br><br>Archives de Neurologie. 1884;6(4):569-76.
Newcombe,<br>Charles F	Epileptiform seizures in general paralysis. 1875;5:198-226.	Case of locomotor ataxy. 1879;2(1):134-8.
Rabagliati,<br>A H	On classification and nomenclature in nervous disorders. 1876;6:27-42.	Critical Digests and Notices of Books. 1879;1(4):529-44.<br><br>Critical Digests and Notices of Books. 1879;2(2):234-50.<br><br>Critical Digests and Notices of Books. 1881;4(1):100-10.<br><br>Critical Digests and Notices of Books. 1881;4(2):233-8.<br><br>Critical Digests and Notices of Books. 1882;5(1):105-9.<br><br>Critical Digests and Notices of Books. 1883;6(3):404-13.<br><br>La psichiatria, la neuropatologia e

		le scienze affine. 1884;6(4):565-7.
Sankey,<br>H R Octavius	A new process for examining the structure of the brain. With a review of some points in the histology of the cerebellum. 1875;5:188-97.	Two cases of microcephalic idiotcy [*sic*] in one family – convulsions of mother during pregnancy. 1878;1(3):391-9.
